# Red-Fleshed Apple Anthocyanin Extracts Attenuate Male Reproductive System Dysfunction Caused by Busulfan in Mice

**DOI:** 10.3389/fnut.2021.632483

**Published:** 2021-06-23

**Authors:** Jihua Xu, Xiang Zhang, Xiaohong Sun, Qiang Lv, Yugang Zhang

**Affiliations:** ^1^Qingdao Key Laboratory of Genetic Development and Breeding in Horticultural Plants, Qingdao Agricultural University, Qingdao, China; ^2^College of Life Sciences, Qingdao Agricultural University, Qingdao, China; ^3^College of Horticulture, Qingdao Agricultural University, Qingdao, China; ^4^School of Pharmacy, Zhejiang Chinese Medical University, Hangzhou, China

**Keywords:** red-fleshed apple anthocyanin extract, busulfan, reproductive system, spermatogenesis, metabolites

## Abstract

In this research, we analyzed the effect of an intragastrical oral administration of red-fleshed apple anthocyanin extract (RAAE) on busulfan-treated mice. First, we showed that the most abundant component in RAAE was cyanidin 3-O-galactoside. To determine the effect of the RAAE, the mice were divided into control and four other different concentrations of RAAE feeding treatment groups (BA0, no RAAE; BA.1, 0.1 mg/kg; BA1, 1 mg/kg; and BA5, 5 mg/kg) following busulfan injection. We observed that RAAE treatments displayed ameliorative effects on male reproductive system dysfunction caused by busulfan, such as recovering the irregular arrangements of seminiferous tubules, increasing the number of spermatogonia and spermatocytes, improving sperm concentration by 3-fold in BA.1, and improving sperm motility by 2-fold in BA1. The liquid chromatography with tandem mass spectrometry (LC-MS/MS) analysis showed significant up- or downregulation of certain metabolites, such as lysophosphatidylcholine (LysoPC), L-arginine, glycine, anandamide, and L-carnitine, which could contribute to the positive effects of RAAE, especially in PBA1 (plasma of BA1) and PBA5 (plasma of BA5). Taken together, the results indicate that 1 mg/kg of RAAE is a suitable concentration for rescuing spermatogenesis in mice. The research suggests that RAAE could be a potential nutraceutical for protecting spermatogenesis after busulfan therapy in cancer.

## Introduction

Busulfan, a chemotherapeutic agent, is widely used in the treatment of malignancies, such as chronic myeloid leukemia. It has the advantages of low cost and could be applied to children under three years of age (1). Meanwhile, its adverse effects have been attracting increasing attention (2–4). In particular, busulfan has the potential to kill spermatogonial stem cells, causing severe damage to the male reproductive system, such as decrease in sperm motility, sperm concentration, and testis weight (5–7). A number of studies have been reported on how to relieve the damage induced by busulfan. Melatonin was found to alleviate busulfan-induced testicular damage by reducing the level of oxidative stress and apoptosis in cells (8). The weight of the testes and spermatogenesis were restored after the administration of genistein in busulfan-treated rats (9). Recently, the use of natural product alginate oligosaccharides for recovering spermatogenesis in busulfan-treated mice has been investigated, and the results have indicated a positive effect of alginate oligosaccharides on the improvement of fertility through the regulation of lipid metabolism homeostasis (1). However, few studies were reported using plant extracts with antioxidant activity for alleviating the damage on the male reproductive system of mice or rats treated with busulfan, other than red ginseng and olive leaf extracts (5, 10).

Anthocyanins belong to flavonoid compounds, a class of plant pigments that widely exist in nature (11). It is well-known that anthocyanins, as effective natural antioxidants, play vital roles in protecting human against diseases, such as cancer, diabetes, and hyperglycemia, and maintaining normal vascular permeability (12). Previous studies that used pomegranate, purple cauliflower, and purple cabbage have reported that anthocyanin extracts have a strong ability to scavenge free radicals, such as hydroxyl, superoxide anions, and 2,2-diphenyl-1-picrylhydrazyl (DPPH) radical (13–15). Recently, much attention has been paid to the use of functional foods rich in anthocyanins because of these beneficial effects (16–18). For example, studies show that supplementing the diet with black rice anthocyanin extract alleviates hepatic steatosis (16), dietary administration of black raspberries inhibits tumor initiation and growth in animal models (17), and phenolics (enriched in anthocyanins) extracted from dark sweet cherry reported as growth inhibitory compounds exhibit anticancer activity (18).

Red-fleshed apple [*Malus sieversii f. neidzwetzkyana* (Dieck) Langenf] is attractive to consumers because of visual skin, red flesh color, high anthocyanin content, and other bioactive phytochemicals (19, 20). Anthocyanin extracts from red-fleshed apple had a strong ability to scavenge DPPH, superoxide anions, and hydroxyl radicals, which further reduced the level of oxidative stress and exhibited stronger antioxidant capacity (11, 21, 22). The previous study on the treatment of porcine granulosa cells with anthocyanins extracted from the mesocarp of red-fleshed apple indicated an alleviating effect on oxidative damage (23). Furthermore, the proliferation of human breast cancer could be inhibited by red-fleshed apple, likely because of its antioxidant and anti-proliferation properties (24). However, all these assays that used red-fleshed apples were performed in *in vitro* studies, and little is known regarding the *in vivo* antioxidant effects of red-fleshed apples. We are particularly interested in the effect of red-fleshed apple anthocyanin extract (RAAE) on busulfan-induced damage in mice. We had previously compared four varieties of red-fleshed apples and found that variety “XJ4” had the highest total phenol and anthocyanin contents with a strong ability to scavenge free radicals (11). Thus, in this study, we set to (a) identify the main compounds in RAAE from “XJ4,” (b) evaluate the efficacy of RAAE on busulfan-induced sterility in mice, and (c) detect the main corresponding metabolites that play roles in rescuing spermatogenesis in mice.

## Materials and Methods

### Red-Fleshed Apple Anthocyanin Extraction

Ripe red-fleshed apple “XJ4s” were collected from the experimental orchard of Qingdao Agricultural University (Qingdao, China). The peel and flesh of “XJ4” were ground into powder in liquid nitrogen using mortar and pestle. Total anthocyanins were extracted with HCl-ethanol (1:999, v/v) under dark conditions at 4°C for 15 h, and the ratio of peel and flesh tissue to extraction buffer was 1:10 (w/v). The extract was evaporated to remove HCl, and the supernatant was filtered through a.45-μm membrane (Sangon Biotech, Shanghai, China) and then concentrated to 3,000 mg/L. The stock was stored at −80°C until use.

### Determination of Total Anthocyanin Content

Total anthocyanin content was assessed using the pH differential method (25). Briefly, the anthocyanin extract (1 ml) was added into a 9-ml sodium acetate buffer (.4 mol/L, pH 4.5) and potassium chloride buffer (.025 mol/L, pH 1), respectively. After incubation at room temperature for 1 h, the absorbance of each sample was spectrophotometrically measured at 510 and 700 nm. Absorbance (A) was calculated according to the following equations:

(1) $$\begin{array}{r} A={\left(A510-A700\right)}_{\text{pH}1}-{\left(A510-A700\right)}_{\text{pH}4.5}, \end{array}$$

Total anthocyanin content (cyanidin 3-O-glucoside equivalents, mg/kg FW) = A × MW × DF / (ε × W), (2).

MW: the molecular weight of cyanidin 3-O-glucoside; DF: dilution factor; ε: the molar absorptivity of cyanidin 3-O-glucoside; and W: fresh weight.

### Identification of Anthocyanin and Proanthocyanidin in Components by Liquid Chromatography With Tandem Mass Spectrometry Analysis

Ground powder, 50 mg, of freeze-dried “XJ4” fruit was dissolved in a 500-μl extracting solution. After vortexing for 10 min, the sample was ultrasonicated for 10 min and then centrifuged at 13,000 rpm at 4°C for 3 min. The above steps were then repeated once. The supernatant was collected and combined, and then transferred into LC vials after filtering (.22-μm pore size, Sangon Biotech, Shanghai, China). The samples were stored at −80°C until the LC-MS/MS analysis.

The samples were analyzed with a UPLC-ESI-MS/MS system (UPLC, Shim-pack UFLC SHIMADZU CBM30A system coupled with Applied Biosystems 6500 Triple Quadrupole). Briefly, the conditions used were (1) ACQUITY UPLC BEH C18 column (2.1 × 100 mm, 1.7 um particle size, Waters Corporation, Milford, MA, USA) and column temperature was 45°C. (2) The A and B mobile phases were water (.1% formic acid) and methanol (.1% formic acid), respectively. (3) The gradient programs were as follows: 0 min, 5% B; 0–6 min, 5–50% B; 6–12 min, 50–95% B; 12–14 min, 95–5% B. (4) The flow rate was.35 ml/min, temperature was 40°C, and injection volume was 2 μl. The effluent was connected to an electrospray ionization (ESI)-triple quadrupole-linear ion trap (QTRAP)-MS. The operation parameters of the ESI source were: ion spray voltage was 5,500 V, source temperature was maintained at 550°C, and curtain gas (CUR) was set at 35 psi.

The anthocyanin and proanthocyanidin components were quantified based on the peak area of analyzed samples and a linear equation of standard curves, according to the following equation:

anthocyanin or proanthocyanidin component content (mg/kg) = c*V/m

C: concentration calculated by peak area of analyzed samples and standard curves (ng/ml); V: volume of solution (μl); m: weight of sample (μg).

### Animals and Diets

The Institute of Cancer Research (ICR) male mice (3 weeks old, weighing 17–18 g) were purchased from Qingdao Institute of Drug Control (Qingdao, China) and raised with a 12-h day/night cycle under controlled laboratory conditions (temperature 23°C and humidity 50–70%). All animal experimental procedures were conducted according to the protocols of the Animal Care and Use Committee of Qingdao Agricultural University [license number: SYXK (SD) 20170005]. A total of 50 mice were randomly divided into five groups (one control and four treatment groups, 10 mice in each group), (1) Control; (2) BA0 (busulfan + deionized water); (3) BA.1 [busulfan +.1 mg/kg body weight (BW)]; (4) BA1 (busulfan + 1 mg/kg BW); and (5) BA5 (busulfan + 5 mg/kg BW). The mice were provided with chow diet and water for 3 days before the administration of anthocyanin extracts. A day before the administration of anthocyanins extract, they were intraperitoneally injected with busulfan (40 mg/kg BW). The mice in the BA.1, BA1, and BA5 groups were fed with anthocyanin extracts at indicated concentrations, while the mice in the BA0 group were fed with an equal amount of deionized water (100 μl). The mice were fed daily for 7 weeks, and their body weight was measured 0, 10, 20, 30, 33, and 35 days after anthocyanin extract administration. On the 35th day, the mice were humanely euthanized, and we used their testis, liver, and kidney weight for further analysis.

### Histological Analysis and Cell Quantification Procedures

The testis, liver, and kidney of the mice were fixed in a formalin solution and then dehydrated in a gradient of ethanol (70, 80, 95, and 100%) before being embedded in paraffin, sectioned at 5 μm thickness, and stained with hematoxylin and eosin according to a standard procedure. The slides were examined under a light microscope (Olympus, Tokyo, Japan) (26). In total, 15 visual fields were randomly examined in each section under the microscope with a 40× objective lens. The number of spermatogonia, spermatocytes, and Leydig was counted using the ImageJ software. Basically, for each sample group, six histologic sections with five fields (three to four seminiferous tubules in each field) in each section were selected. In each field, all intact cross-section seminiferous tubules were used for investigation. The number of spermatogonia and spermatocytes was calculated by dividing the total number of each type germ cell by the total number of seminiferous tubules examined. Leydig cells were arranged in clusters and adjacent to seminiferous tubules. In this study, a whole group of Leydig cells was counted, and the number of Leydig cells was quantified as the average number per field.

### Terminal dUTP Nick-End Labeling Staining

Apoptosis analysis was conducted using the One Step TUNEL Apoptosis Assay Kit (Cat# C1086, Beyotime Biotechnology, Shanghai, China). Tissue sections were under deparaffinization first: soaked in xylene for three changes with 5 min each time, followed by rehydration in 100, 90, and 70% ethanol for 5 min, and then washed with distilled water for 5 min. Then, the sections were treated with proteinase K (DNase free, 20 μg/ml) and incubated at 37°C for 15 min. After five times of washing with phosphate-buffered saline (PBS), the sections were incubated with a TUNEL reaction mixture at 37°C for 90 min in the dark, and then washed again with PBS three times. Finally, the sections were stained with Hoechst33342 (C1022, Beyotime Biotechnology, Shanghai, China) for 10 min, washed with PBS three times, and then observed immediately with an Evos FL auto 2 imaging system (Invitrogen, Carlsbad, CA, United States). The cell number and TUNEL-positive cells in seminiferous tubules were counted using ImageJ. For each sample group, five histologic sections with five fields in each section were selected. TUNEL-positive cells (%) were calculated by dividing the number of positive cells by the number of total cells in intact cross-section seminiferous tubules per field.

### Analysis of Spermatozoa Motility

On the 35th day, caudal epididymides were extracted and placed in an RPMI medium, finely chopped, and then the spermatozoa were stained with Eosin Y (1%) as described previously by Shin et al. (27). Spermatozoa motility was evaluated through a computer-assisted sperm assay (CASA). The evaluation procedure is based on WHO guidelines (1, 28). Briefly, the spermatozoa were suspended in the medium with Dulbecco's modified Eagle medium/F-12 nutrient mixture (DMEM/F12) and 10% fetal bovine serum (FBS), incubated for 30 min at 37.5°C, and then placed in a counting chamber. The motility was observed with a sperm class analyzer (CASA; MICROPTIC SL, Barcelona, Spain). The four classes of sperm motility were (a) rapid progressive (linear velocity > 22 μm/s), (b) slow progressive (linear velocity < 22 μm/s and curvilinear velocity > 5 μm/s), (c) non-progressive (curvilinear velocity < 5 μm/s), and (d) immotility.

### Plasma Sample Preparation and Liquid Chromatography With Tandem Mass Spectrometry Analysis

The mice were sacrificed under diethyl ether anesthesia, and a 500-ul blood sample from each mouse was withdrawn by cardiac puncture using syringes. The blood samples were immediately centrifuged at 3,000 rpm for 15 min, and then 100 μl supernatant plasma samples were collected and immediately stored at −80°C. For the LC-MS/MS analysis, a total of 20 μl internal standard (10 μl of 2-chloro-l-phenylalanine in methanol, 0.3 mg/m, and 10 μl of LysoPC 17:0, 0.01 mg/ml) was added into a 100-μl plasma sample. Then, 300 μl of methanol and acetonitrile (2:1, V/V) was subsequently added into the mixture. The sample was ultrasonicated for 10 min after vortexing for 1 min. Then, the mixture was stored at −20°C for 30 min and subsequently centrifuged at 13,000 rpm (4°C) for 10 min. Then, 300 μl supernatant was collected in a brown glass vial, dried in centrifugal freeze-drying equipment (LNG-T98, Huamei Instrument, Taicang, China), and then dissolved in 400 μl methanol and water (1:4, v/v). The samples were vortexed for 30 s, ultrasonicated for 2 min, and then centrifuged at 13,000 rpm (4°C) for 10 min. The supernatant was filtered through a 0.22-μm membrane before the LC-MS/MS analysis.

The samples were analyzed using an ACQUITY UPLC system (Nexera UPLC, Shimadzu, Tokyo, Japan) coupled with AB Sciex Triple TOF 5600 (LC/MS) as reported (29). Briefly, a total of 10-μl samples were injected into the ACQUITY UPLC BEH C18 column (2.1 × 100 mm, 1.7 μm particle size, Waters Corporation, Milford, MA, United States), with a column temperature of 45°C. The A and B mobile phases were water/formic acid (99:1, v/v) and water/acetonitrile (99:1, v/v), respectively. The gradient used was as follows: 0–1.5 min, 5% B; 1.5–3 min, 5–30% B; 3–7 min, 30–60% B; 7–9 min, 60–90% B; 9–11 min, 90–100% B; 11–13 min, 100% B; 13–13.2 min, 100–5% B; 13.2–16 min, 5% B.

The resulting LC-MS/MS raw data were analyzed using the Progenesis QI v2.3 software (Waters Corporation, Milford, MA, USA) following the parameters previously described (30). The metabolites were identified with Progenesis QI v2.3 using both public databases (http://www.hmdb.ca/; https://www.lipidmaps.org/) and self-built databases by Lu-Ming Biotech (Shanghai, China). The differentially expressed metabolites in four group comparisons, namely, plasma of BA0 (PBA0) vs. control, plasma of BA.1 (PBA.1) vs. control, plasma of BA1 (PBA1) vs. control, and plasma of BA5 (PBA5) vs. control were conducted. The thresholds of variable importance in projection (VIP) > 1 and *p* < 0.05 (two-tailed Student's *t*-test) were considered as significantly different metabolites.

### Statistical Analysis

The experimental data were presented as means ± SE. One-way analysis of variance (ANOVA) and multiple comparisons were performed to determine significant differences among the different groups. GraphPad Prism software 5.0 (San Diego, CA, USA) and Origin software 9.0 (Northampton, MA, USA) were used to process data and plot charts.

## Results

### Phenotypic Characters and Anthocyanin and Proanthocyanid in Component Contents of Red-Fleshed Apple

**Figure 1:**
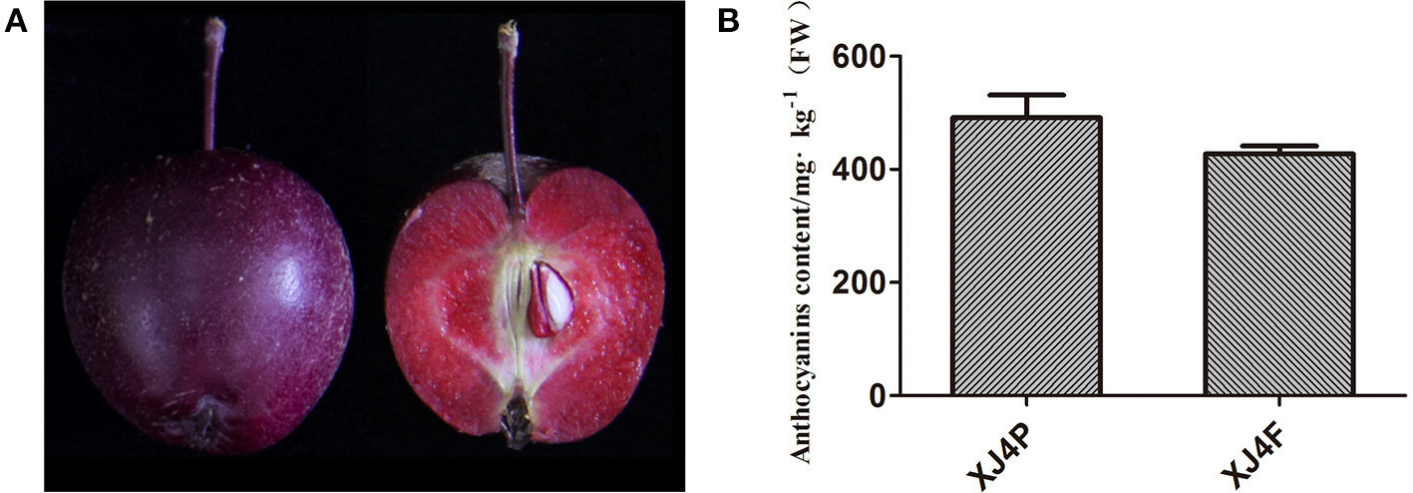
Phenotypic characteristics and anthocyanin content of red-fleshed apple variety “XJ4.” **(A)** Intact fruit and longitudinal section of “XJ4” to show peel and flesh. **(B)** Anthocyanin content of “XJ4” peel and flesh. XJ4P and XJ4F represent peel and flesh of “XJ4,” respectively.

The mature fruit of red-fleshed apple variety “XJ4” has purple red peel and dark red flesh (Figure 1A). The total anthocyanin content in the peel and the flesh was 491.3 mg/kg FW (XJ4P) and 427.3 mg/kg FW (XJ4F), respectively, as determined by the pH differential method, with no significant difference (Figure 1B). Therefore, the RAAE from the whole fruit was used in this study. It was concentrated first and then diluted into 0.1, 1, and 5 mg/kg BW for oral administration. We further identified the specific components of anthocyanin and proanthocyanidin in the “XJ4” whole fruit by LC-MS/MS. In total, 14 anthocyanins and four B-type proanthocyanidins (procyanidin B1, procyanidin B2, procyanidin B3, and procyanidin C1) were identified, with cyanidin 3-O-galactoside being the most abundant (Table 1). Cyanidin 3-O-galactoside was found to be 179.810 mg/kg, followed by procyanidin C1 (14.564 mg/kg), procyanidin B2 (10.474 mg/kg), cyanidin 3-O-arabinoside (6.489 mg/kg), and cyanidin 3-O-glucoside (2.990 mg/kg) (Table 1). The proportion of cyanidin 3-O-galactoside was over 80% compared with the total content (218.149 mg/kg) of all 18 anthocyanin and proanthocyanidin components (Table 1).

**Table 1:** Analysis of anthocyanin and proanthocyanidin compounds in RAAE.

**Number**	**Identification**	**Molecular** **formula**	**Precursor ions** **[M]^+^ or[M + H]^+^(m/z)**	**Product ions (m/z)**	**Theoretical mass (m/z)**	**Retention time (min)**	**Calculated content (mg/kg)**
1	Delphinidin 3-O-rutinoside	C_27_H_31_O_16_	611.1	303.2	611.1612	5.92	0.006
2	Cyanidin 3-O-galactoside	C_21_H_21_O_11_	449.1	287	449.1084	5.78	179.810
3	Cyanidin 3-O-arabinoside	C_20_H_19_O_10_	419.1	287.1	419.0978	6.75	6.489
4	Cyanidin 3-O-glucoside	C_21_H_21_O_11_	449.2	287.1	449.1084	6.34	2.990
5	Cyanidin 3-O-rutinoside	C_27_H_31_O_15_	595.2	287.1	595.1663	6.96	0.005
6	Cyanidin 3-O-(6-O-malonyl-beta-D-glucoside)	C_24_H_23_O_14_	535.1	287.1	535.1088	8.99	0.051
7	Pelargonidin 3-O-galactoside	C_21_H_21_O_10_	433.2	271.1	433.1135	6.65	1.130
8	Pelargonidin 3-O-arabinoside	C_20_H_19_O_9_	403.1	271.1	403.1029	7.63	0.009
9	Pelargonidin 3-O-glucoside	C_21_H_21_O_10_	433.2	271.1	433.1135	7.33	0.007
10	Pelargonidin 3-O-rutinoside	C_27_H_31_O_14_	579.1	271.1	579.1708	8.11	0.002
11	Peonidin 3-O-galactoside	C_22_H_23_O_11_	463.3	301.2	463.1240	7.47	1.444
12	Peonidin 3-O-arabinoside	C_21_H_21_O_10_	433.2	301.1	433.1135	8.44	0.062
13	Peonidin 3-O-glucoside	C_22_H_23_O_11_	463.3	301.2	463.1240	8.01	0.068
14	Malvidin 3-O-glucoside	C_23_H_25_O_12_	493.1	331.2	493.1346	8.50	0.007
15	Procyanidin B1	C_30_H_26_O_12_	579.1	427.1	578.1424	2.61	0.936
16	Procyanidin B2	C_30_H_26_O_12_	579.1	427.1	578.1424	4.00	10.474
17	Procyanidin B3	C_30_H_26_O_12_	579.1	427.1	578.1424	2.41	0.095
18	Procyanidin C1	C_45_H_38_O_18_	889.2	601.0	866.2058	5.21	14.564

### Effects of Red-Fleshed Apple Anthocyanin Extract on Weight of Mice Injected With Busulfan

**Figure 2:**
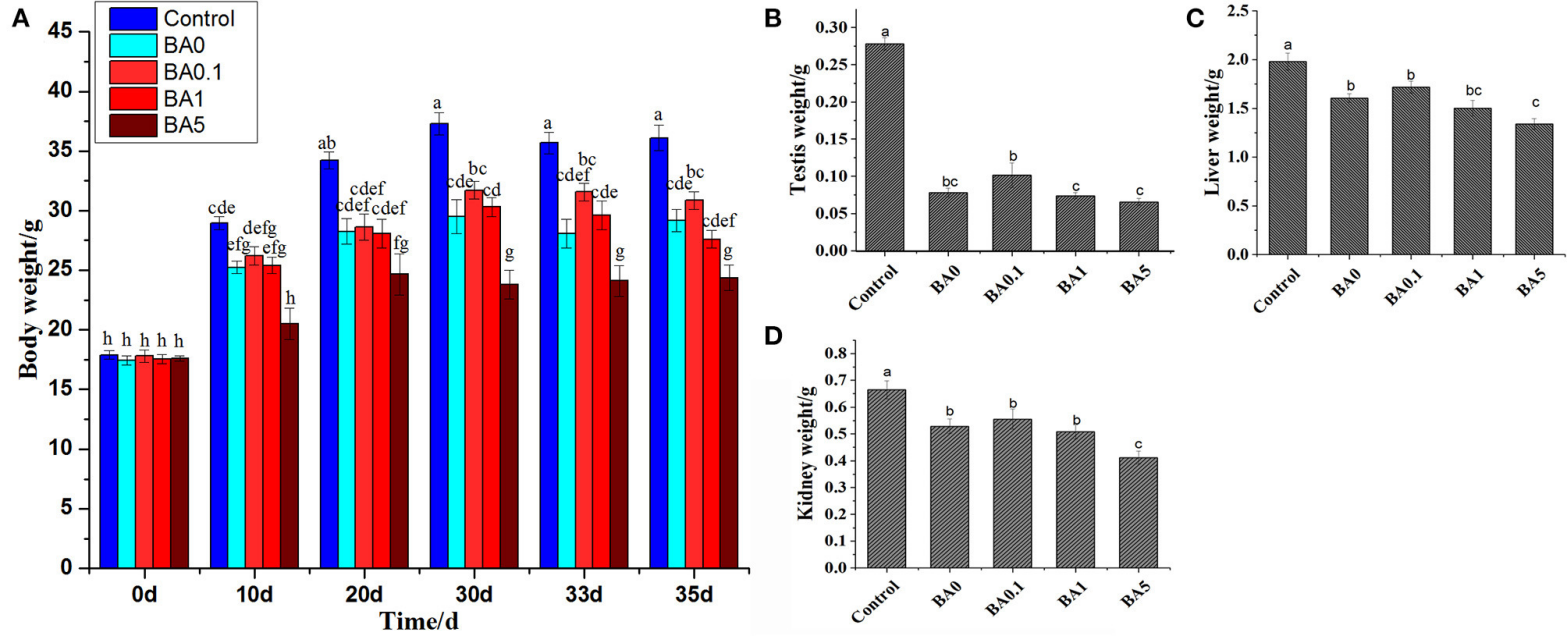
The body and organ weights of mice. **(A)** Body weight of mice. **(B)** Testis weight. **(C)** Liver weight. **(D)** Kidney weight. Within the samples, values with different letters (a, b, c) are significantly different (*p* < 0.05). Body weight was measured 0, 10, 20, 30, 33, and 35 days after anthocyanin extract administration. The weights of testis, liver, and kidney were measured 35 days after anthocyanin extract administration.

Before the treatment, the body weight of mice was consistently around 17–18 g, and the differences began to appear after 10 days of anthocyanin extract administration, as shown in Figure 2A. Gradually, the side effect of busulfan on body weight appeared. For example, on the 10th day, there was no significant difference in body weight between the control mice and the mice in the BA0 group, but on the 20th, 30th, and 33rd days, the body weight of the BA0 group mice was significantly lower than that of the control group mice. However, the administration of RAAE did not significantly alleviate the decreased body weight brought about by busulfan. In contrast, the body weight of the mice in the highest anthocyanin concentration group (BA5) significantly decreased compared with that of the mice in the BA0 group. This indicated the severe damage caused by high concentration of the anthocyanin extract. Similarly, in terms of the testis (Figure 2B), liver (Figure 2C), and kidney (Figure 2D), there was no significant increase in body weight after the RAAE treatment, and the lowest body weight was also observed in the BA5 group mice.

### Effects of Red-Fleshed Apple Anthocyanin Extract on Busulfan-Induced Damage to Different Tissues

**Figure 3:**
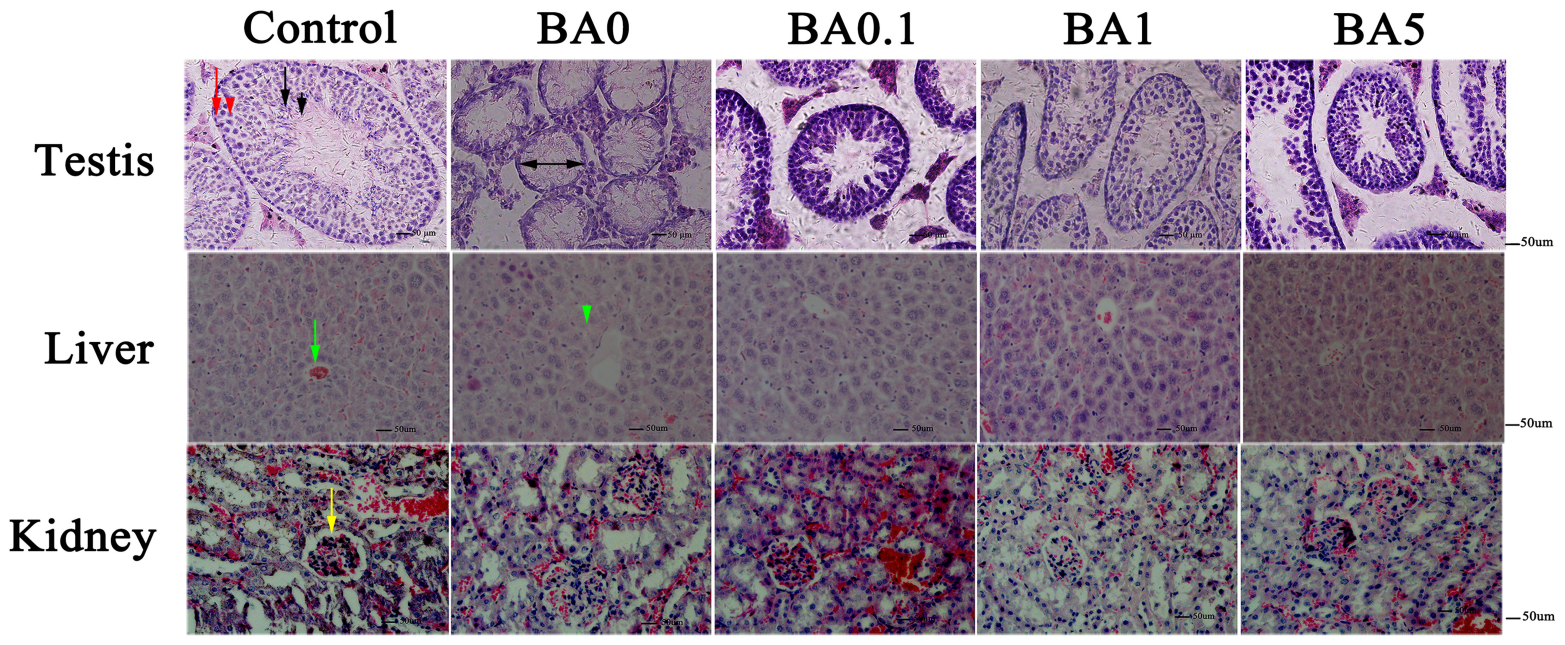
Effect of RAAE on busulfan-induced histological damage in different organs. In the first panel, the testis in the control group showed normal seminiferous tubules. Red arrow indicates spermatogonia, red arrowhead indicates primary/secondary spermatocytes, black arrow represents spermatids, and black arrowhead represents spermatozoa. The diameter of the seminiferous tubules is shown by black two-headed arrows. In the second panel, the central vein is indicated with a green arrow, and cell necrosis is shown by a green arrowhead. In the third panel, glomerulus with abundant capsular space is indicated by a yellow arrow.

**Figure 4:**
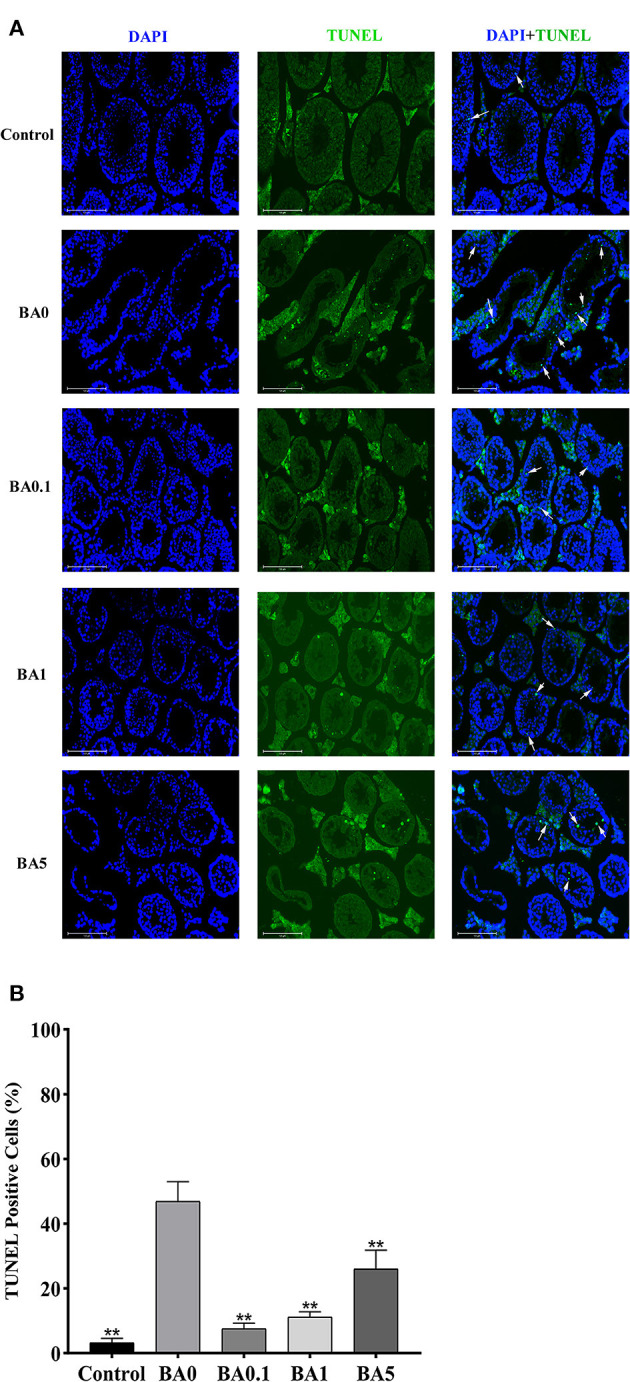
Cell apoptosis was detected by TUNEL assay in the seminiferous tubules in the control and different treatment groups. **(A)** The representative images of TUNEL staining. Blue means nuclei stained with DAPI, while green means TUNEL-positive cells stained with TUNEL, white arrows show apoptotic cells, scale bar = 125 μm. **(B)** The percentage of TUNEL-positive cells. TUNEL-positive cells (%) were calculated by the number of positive cells divided by the total number of cells **p < 0.01.

The effects of RAAE on the recovery of seminiferous tubules damaged by busulfan were investigated. Regular seminiferous tubules (normal spermatogonia, primary/secondary spermatocytes, spermatids, and spermatozoa in seminiferous tubules) were observed in control as shown by histological structure (Figure 3). However, after the busulfan treatment, irregular seminiferous tubule arrangements and large vacuoles were found, which indicated the severe damage caused by the busulfan treatment. In addition, after the busulfan treatment, the diameter of the seminiferous tubules was obviously reduced compared with control. As shown in Figure 3, for the BA.1, BA1, and BA5 groups, RAAE could significantly alleviate the irregular arrangements and increase the diameter of the seminiferous tubules. We further quantitated the related parameters in seminiferous tubules in the control and treatment groups (Table 2). After the busulfan treatment, the number of spermatogonia, spermatocytes, and Leydig cells, and the diameter of seminiferous tubules were significantly decreased (BA0, *p* < 0.01); while in BA.1 and BA1, the value of all these four parameters was significantly increased when compared with BA0 (*p* < 0.01). However, in BA5, changes in the number of spermatogonia and Leydig cells were not significant when compared with BA0 (Table 2). In order to further verify the effect of RAAE on the recovery of seminiferous tubules caused by busulfan, the TUNEL assay was used to detect cell apoptosis in the control and treatment groups (Figure 4). There were few apoptotic cells observed in the seminiferous tubules of the control group, while in BA0, a number of apoptotic cells were observed (Figure 4A), significantly higher than in control (*p* < 0.01; Figure 4B). However, in BA.1, BA1, and BA5, the number of apoptotic cells was gradually reduced when compared with BA0 (*p* < 0.01; Figure 4B).

**Table 2:** Quantification of related parameters in seminiferous tubules.

**Group**	**Control**	**BA0**	**BA0.1**	**BA1**	**BA5**
Spermatogonia	37.86 ± 2.55^**^	19.57 ± 2.17	35.14 ± 2.09^**^	31.43 ± 1.45^**^	25.29 ± 2.30
Spermatocytes number	148.29 ± 8.96^**^	49.71 ± 5.28	112.29 ± 8.80^**^	93.43 ± 8.88^**^	68.71 ± 4.53^*^
Leyding cells number	20.57 ± 1.25^**^	8.29 ± 0.61	15.14 ± 1.22^**^	12.43 ± 0.72^**^	10.43 ± 0.78
Seminiferous tubules diameter	204.55 ± 8.05^**^	103.86 ± 3.08	177.95 ± 9.23^**^	151.59 ± 5.38^**^	125.45 ± 2.13^**^

*Within the samples, significant differences*

* *p < 0.05 and*

** *p < 0.01, respectively. The data were presented as mean ± SD*.

In addition to the testis, we further analyzed the effects of busulfan on the liver and kidney without or with RAAE. In the liver, normal liver lobular structure was observed in control, and the cells around the central vein were organized tightly and in order; while in the BA0 group, the cells showed apoptosis, cytoplasmic vacuolation, and condensed nuclei (Figure 3). The RAAE treatment groups, on the other hand, showed a lack of apoptotic cells around the central vein. This observation was similar to the results reported regarding the effects of quercetin 7-rhamnoside on CCl_4_-damaged mice (31). In the kidney, there were no obvious histological changes among the control and four different treatment groups (Figure 3). The structure of glomerulus was clear with an abundant capsular space in all groups. Compared with the testis, the damaging effects of busulfan on the liver and kidney appeared milder, especially on the kidney.

### Effects of Red-Fleshed Apple Anthocyanin Extract on Busulfan-Induced Damage to Sperm Concentration and Sperm Motility

**Figure 5:**
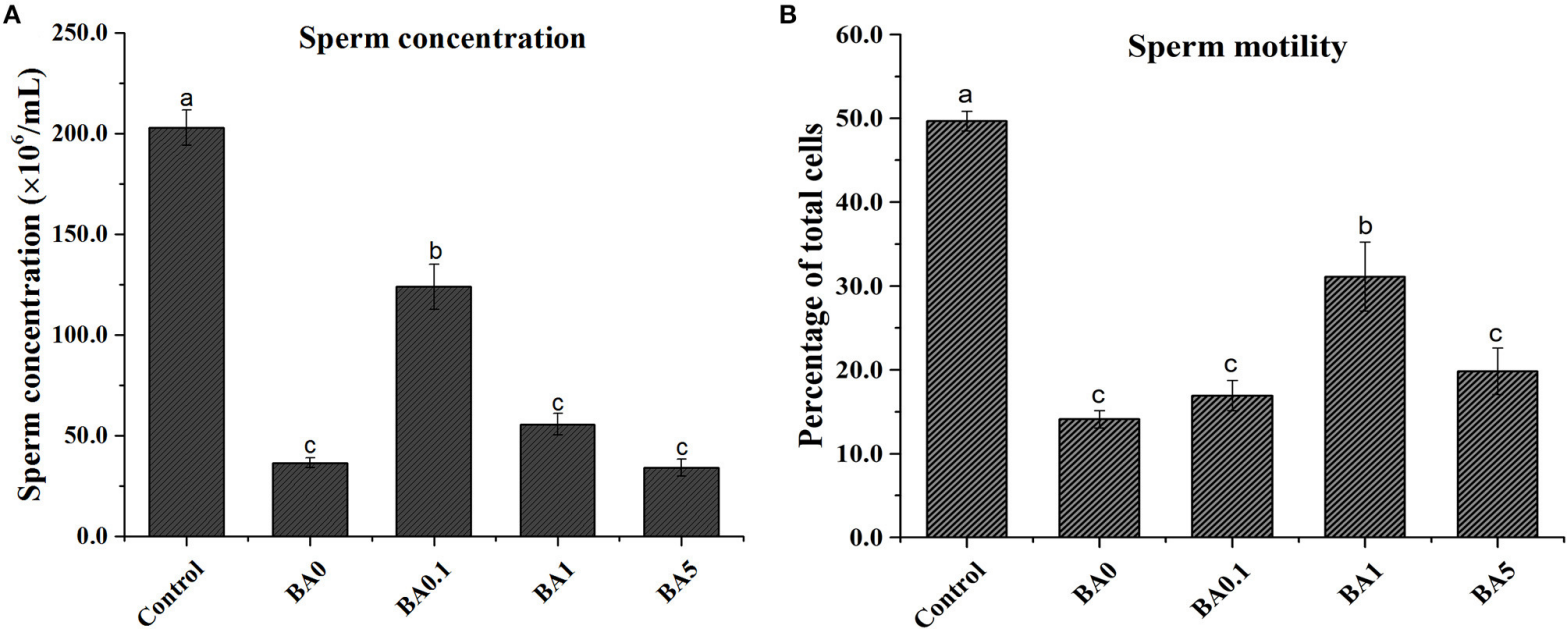
Effect of the anthocyanin extract from red-fleshed apple on **(A)** sperm concentration and **(B)** sperm motility of mice after injection of busulfan. Within the samples, values with different letters (a, b, c) indicate significant differences (*p* < 0.05).

As shown in Figure 5, compared with control, the busulfan treatment (BA0) group exhibited dramatically reduced sperm concentration (from 203 to 37 million/ml) and motility (from 49.7 to 14.1%). After the administration of different concentrations of RAAE (BA.1, BA1, and BA5), BA.1 significantly ameliorated the sperm concentration by more than 3-fold compared with BA0 (Figure 5A). Similarly, BA1 could significantly improve the sperm mobility by more than 2-fold compared with BA0 (Figure 5B). Interestingly, the group with the highest concentration of RAAE (BA5) did not show clear alleviation of the busulfan-induced damage to sperm concentration or motility.

### Effects of Red-Fleshed Apple Anthocyanin Extract on Plasma Metabolism

**Figure 6:**
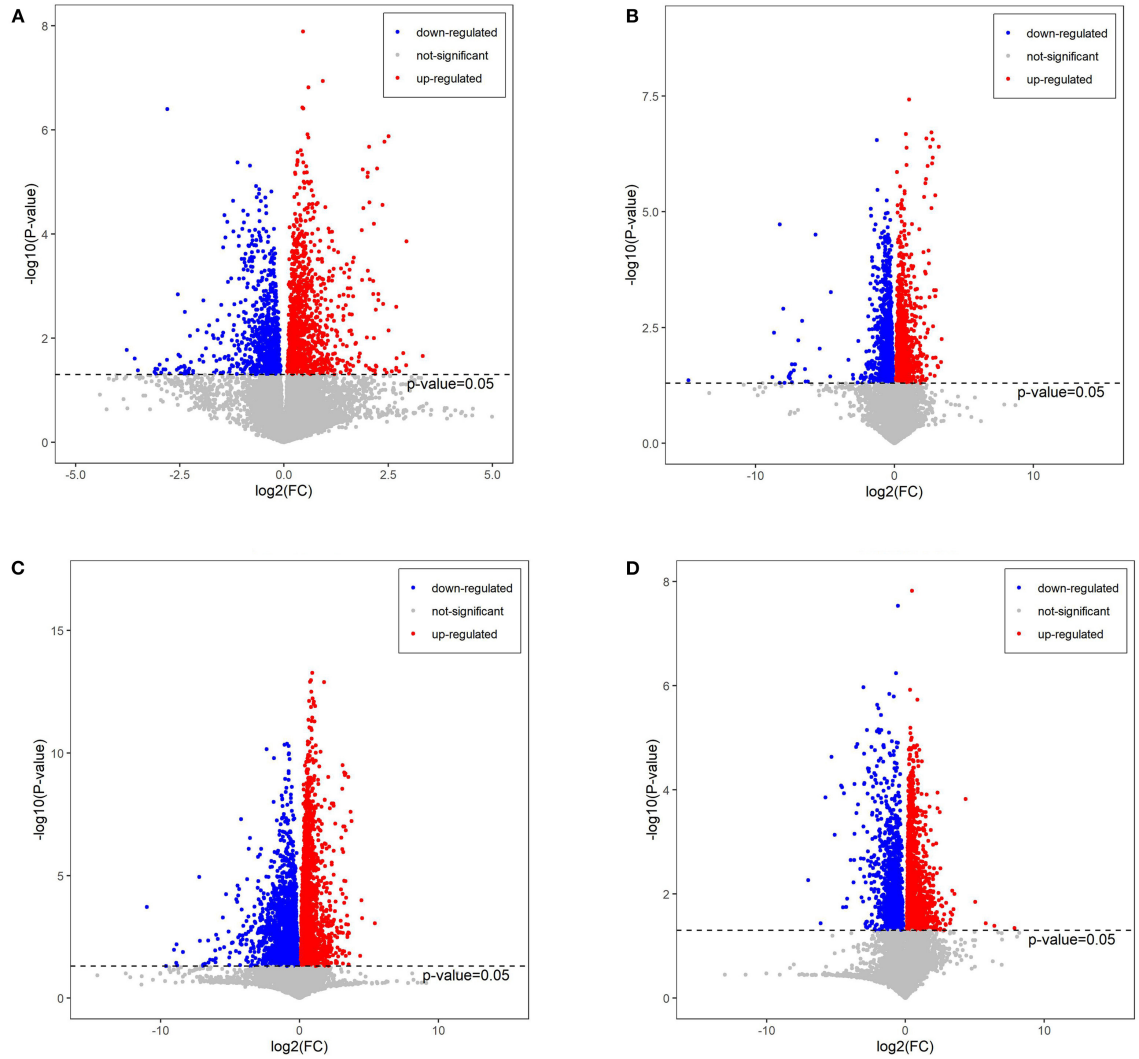
Volcano plot of metabolites in different combinations. **(A)** PBA0 vs. control. **(B)** PBA.1 vs. control. **(C)** PBA1 vs. control. **(D)** PBA5 vs. control.

The plasma was analyzed in order to understand the metabolite changes *in vivo* after the RAAE intake in the mice. There were 118 significantly different metabolites detected in PBA0 vs. control, with 62 metabolites upregulated and 56 metabolites downregulated. After the administration of different concentrations of RAAE (BA.1, BA1, and BA5), the number of significantly different metabolites increased to 128, 193, and 202 in PBA.1 vs. control, PBA1 vs. control, and PBA5 vs. control, respectively (Supplementary Table 1, Figure 6), in which 38, 53, and 75 metabolites showed upregulation, respectively, whereas 90, 140, and 127 metabolites showed downregulation, respectively.

**Figure 7:**
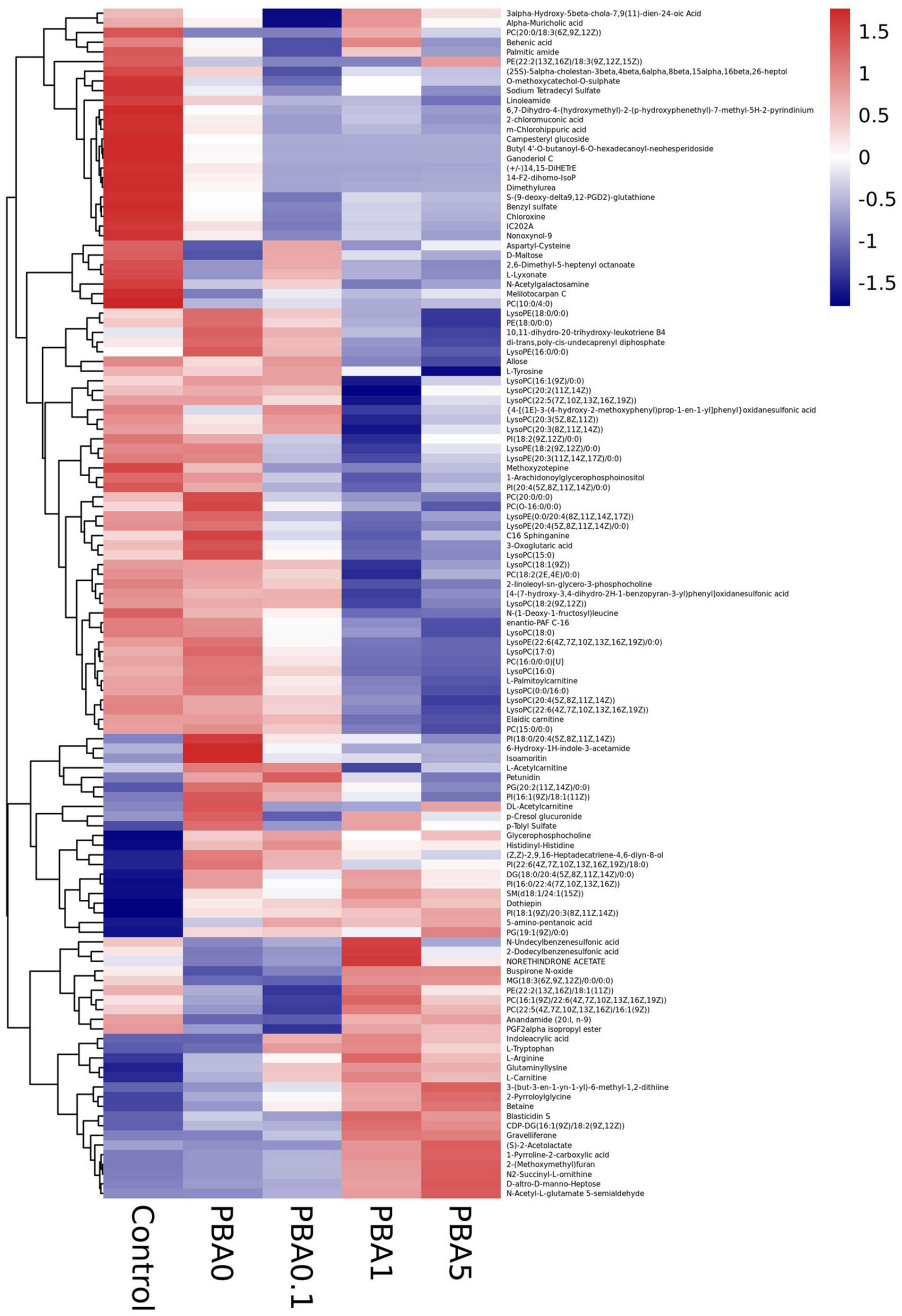
Heat map analysis of metabolites in different treatment groups.

The top 50 significantly altered metabolites were collected from each comparison for further study. It was observed that in the control group there were more upregulated and less downregulated metabolites, and that the busulfan treatment decreased the number of upregulated metabolites (Figure 7). Interestingly, the number of downregulated metabolites was gradually increased, with the increase in the concentration of RAAE. This observation was consistent with the tendency of all significantly different metabolites (Supplementary Table 1). The main downregulated metabolites were LysoPC and lysophosphatidyl ethanolamine (LysoPE) in PBA.1, and especially in PBA1 and PBA5. On the other hand, we detected the upregulation of betaine and amino acids, such as L-arginine, L-carnitine, and 2-pyrroloylglycine, after the RAAE treatment, especially in the PBA1 and PBA5 groups. Further, while the anandamide level decreased after the busulfan treatment, it increased to the level of control in the PBA1 and PBA5 groups (Figure 7). These observations indicated that metabolites, such as LysoPC, LysoPE, betaine, L-arginine, L-carnitine, 2-Pyrroloylglycine, and anandamide, might play important roles in alleviating the damage caused by busulfan in mice.

### Kyoto Encyclopedia of Genes and Genomes Pathway Analysis

**Figure 8:**
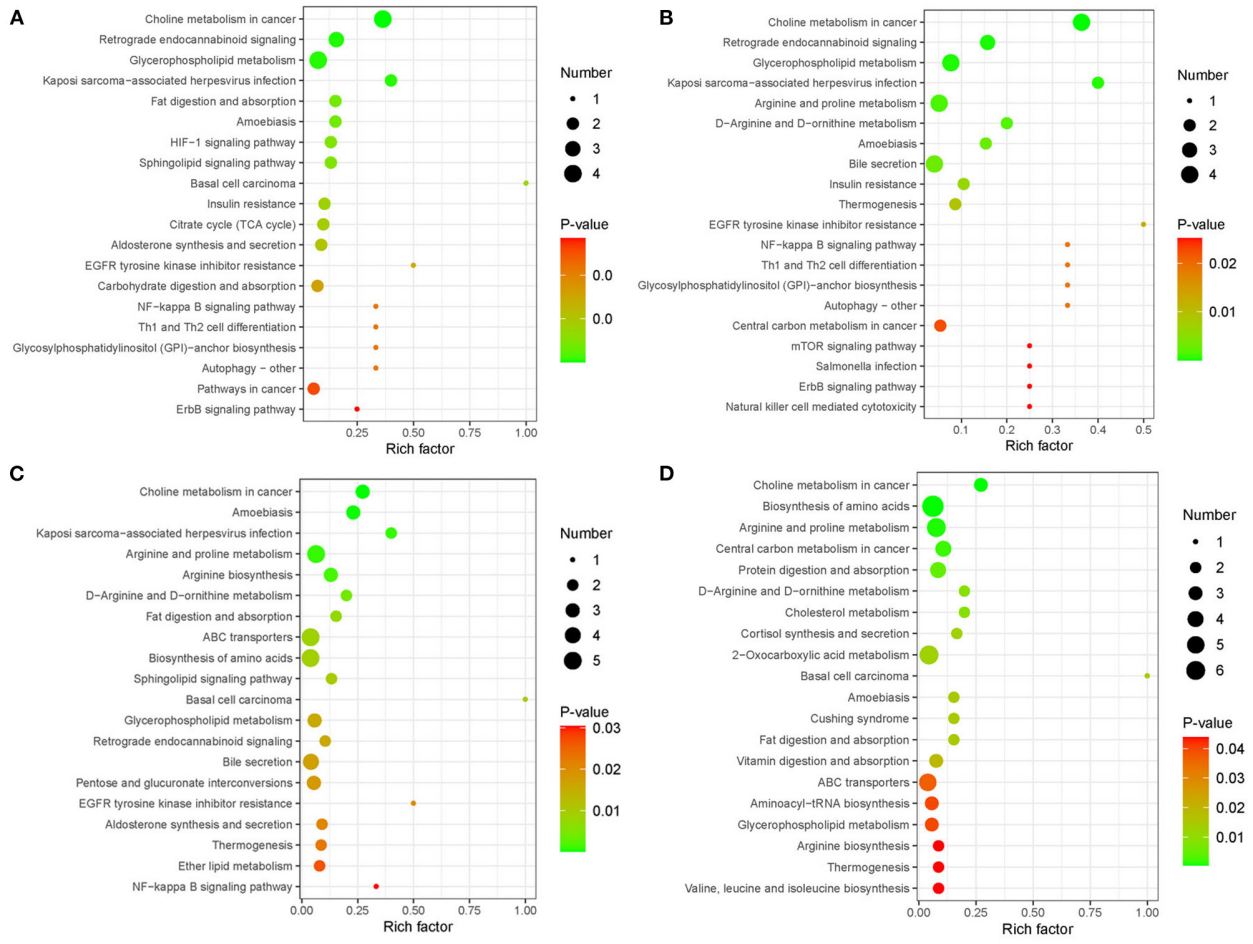
Kyoto Encyclopedia of Genes and Genomes (KEGG) enrichment analysis of metabolic pathway in different combinations. **(A)** PBA0 vs. control. **(B)** PBA.1 vs. control. **(C)** PBA1 vs. control. **(D)** PBA5 vs. control.

We further studied the metabolic pathway enrichment of the significantly different metabolites in all four group comparisons (PBA0 vs. control, PBA.1 vs. control, PBA1 vs. control, and PBA5 vs. control), using differentially expressed metabolites mapped to the KEGG database (http://www.kegg.jp/). In PBA0 vs. control, the largest three groups of significantly different metabolites were in choline metabolism in cancer, retrograde endocannabinoid signaling, and glycerophospholipid metabolism (Figure 8A). Notably, the significantly different metabolites in the treatment group comparisons PBA.1 vs. control, PBA1 vs. control, and PBA5 vs. control were mainly enriched in choline metabolism in cancer, glycerophospholipid metabolism, arginine and proline metabolism, D-arginine and D-ornithine metabolism, amoebiasis, and thermogenesis (Figures 8B–D). The number of metabolites in choline metabolism in the cancer category was gradually reduced with the increase in RAAE concentration.

**Figure 9:**
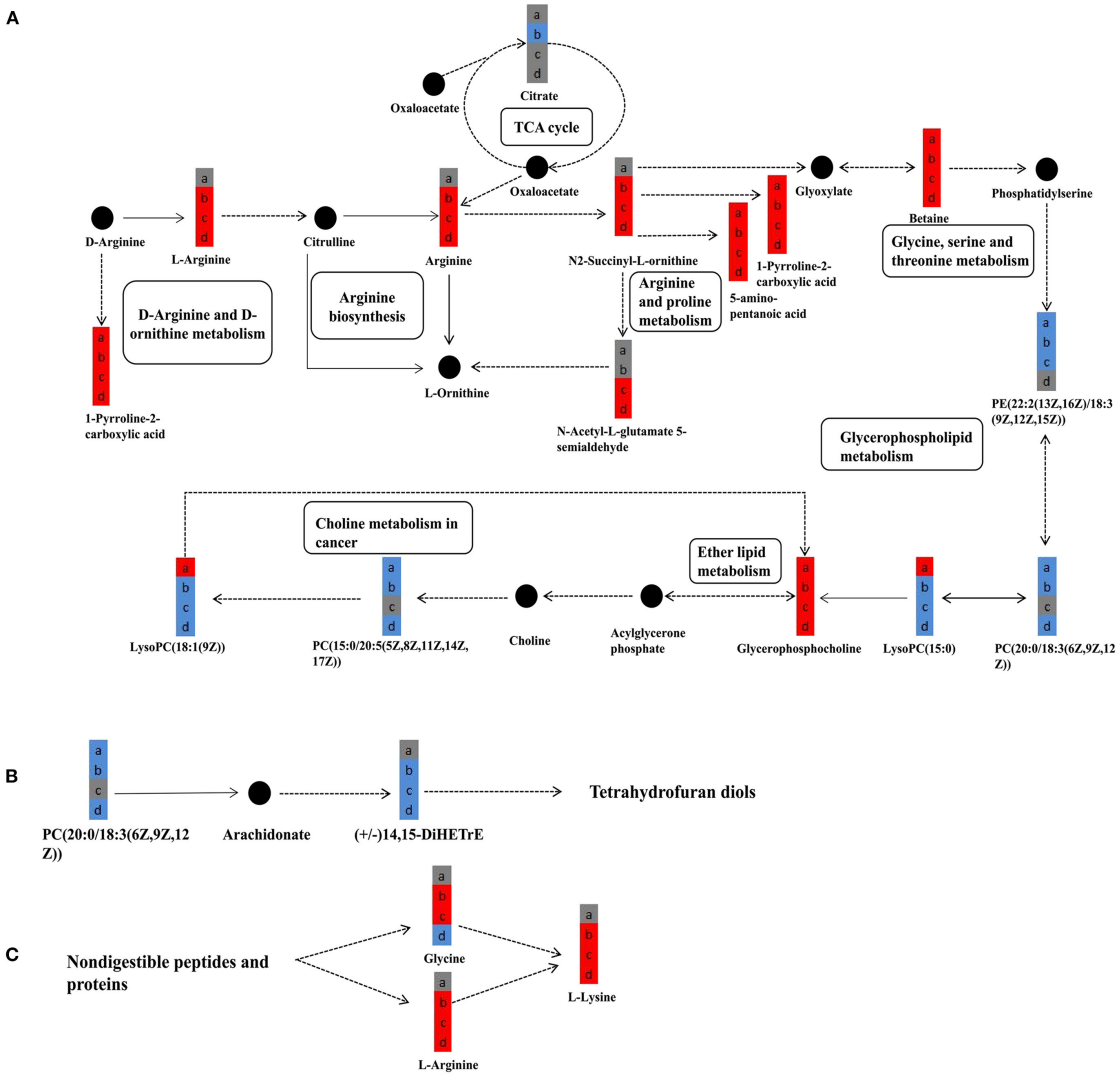
Significantly different metabolites in metabolic pathways in different combinations. **(A)** The major metabolic pathways of significantly different metabolites. **(B)** Tetrahydrofurandiols pathway. **(C)** Lysine-related pathway. a, b, c, and d represent PBA0 vs. control, PBA.1 vs. control, PBA1 vs. control, and PBA5 vs. control, respectively. Solid and dashed lines represent direct and indirect procedure, respectively. Red and blue colors represent upregulation and downregulation, respectively, and gray color represents no significant change. Filled circles with black color represent metabolites in the pathway but undetected in the study.

The further analysis found that the levels of L-arginine, arginine, and N2-Succinyl-L-ornithine were significantly upregulated after the RAAE treatment (Figure 9A), while the levels of LysoPC (15:0) and LysoPC [18:1(9z)] were significantly downregulated (Figure 9A). Furthermore, RAAE could inhibit the biosynthesis of tetrahydrofurandiols by decreasing (+/–) 14,15-DiHETrE, as shown in the treatment group comparisons PBA.1 vs. control, PBA1 vs. control, and PBA5 vs. control (Figure 9B). In contrast, RAAE could promote the synthesis of lysine, as shown in Figure 9C.

## Discussion

Red-fleshed apples are a functional fruit and are preferred by consumers and breeders not only because of the appealing red color of both their skin and flesh but also because of the large amount of natural antioxidant compounds, such as anthocyanins, in both their skin and flesh (22, 23, 32, 33). Anthocyanins have been found to help prevent diseases, such as cancer, diabetes, and hyperglycemia (34). Studies have shown that while busulfan is an effective anti-cancer drug, it could cause severe damage to the male reproductive system in humans (5–7). In this study, we have demonstrated an ameliorative effect of RAAE on male reproductive system dysfunction following exposure to busulfan in mice, pointing out the potential use of RAAE in developing drugs to assist spermatogenesis after cancer therapy.

It was observed that the busulfan treatment resulted in irregular seminiferous tubule arrangements and decreased seminiferous tubule diameter. Based on a previous study (35), spermatogenic cell loss might be the main factor for the decreased diameter. The RAAE treatment could reduce the damage caused by busulfan on seminiferous tubules based on histological analysis (Figure 3) and TUNEL assay (Figure 4). The findings were in line with previous research that used genistein, melatonin, L-carnitine, and molybdenum in murines (9, 36–38). Similarly, plant extracts containing natural antioxidant and phenolic compounds, such as olive leaf and Korean red ginseng, were reported to have positive effects on attenuating the testis dysfunction induced by busulfan (5, 10). While previous studies have mainly focused on the side effect of busulfan on the male reproductive system, in this study, we have also investigated the effect of busulfan on the liver and kidney. The results indicated that the liver and kidney were not as sensitive as testis to the busulfan treatment in mice, and that the RAAE treatment could reduce the damage caused by busulfan. Early studies also reported histological changes induced by busulfan in the lymphoid and gastrointestinal tissues of adult rats (39, 40). In contrast to a previous study that showed increased testes weight after the administration of genistein in busulfan-treated rats (9), we did not find significant amelioration in the weight of the testes, liver, and kidney as well as body weight.

Generally, the data did not show a dose-dependent trend for the alleviation of busulfan-induced damage by the RAAE treatment. These results are in agreement with plant extract studies on busulfan-induced damage that used olive leaf and Korean red ginseng (5, 10). However, based on the results of body and organ weights, busulfan plus the highest concentration of RAAE (BA5) caused negative effects (Figure 2), whereas busulfan plus lower concentrations of RAAE (BA.1 and BA1) ameliorated sperm concentration and mobility by more than 3-fold and more than 2-fold compared with busulfan alone (BA0), suggesting that BA.1 and BA1 might be more appropriate for rescuing spermatogenesis in mice (Figure 5). In this regard, a previous study has also indicated that the higher dose of olive leaf extract had an adverse effect on rat liver tissue (41).

The analysis showed the anthocyanin content in the peel and flesh of red-fleshed “XJ4” to be over 400 mg/kg FW (Figure 1), which is much higher than the anthocyanin content of typical commercial white-fleshed cultivar “Fuji” by about 8 mg/kg FW (24). It has been suggested that the damage induced by busulfan to the spermatogenesis is mainly due to oxidative stress and lipid peroxidation (5, 6, 42). The main reason for RAAE to reduce the damage induced by busulfan is high anthocyanin content, which could scavenge free radicals and thus reduce ROS production and lipid peroxidation. Since RAAE is a mixture of various components, it is unclear which bioactive ingredient (s) in anthocyanins played the role in alleviating the adverse effects induced by busulfan. In this regard, we identified cyanidin 3-O-galactoside as the most abundant anthocyanin, consisting of what was observed in other red-fleshed apples by previous studies (24, 43). It will be interesting to examine whether cyanidin 3-O-galactoside might be the active component that is able to reduce the adverse effect caused by busulfan in mice.

From the analysis of plasma metabolism, we found that LysoPC was downregulated in PBA.1, PBA1, and PBA5. A previous study that used alginate oligosaccharides to rescue busulfan-induced testis damage also found that many lipids were changed after alginate oligosaccharides treatment (1). Lipid metabolism has been suggested to play important roles in spermatogenesis and male fertility (44, 45). LysoPC, which resulted from the hydrolytic cleavage of phosphatidylcholine (PC), is the primary component of low-density lipoprotein (46), and it can bind to G protein-coupled and Toll-like receptors to activate downstream signaling pathways and play important biological roles, such as increasing pro-inflammatory cytokines, inducing oxidative stress, and producing apoptosis (46, 47), in organisms. Thus, the downregulation of LysoPC, which was observed in the RAAE treatment groups, could contribute to the reduction of damage caused by busulfan.

It is interesting to note the significant upregulation of L-arginine and L-lysine in all the RAAE treatment groups, and the upregulation of glycine in the PBA.1 and PBA1 groups. According to several studies, arginine is essential for promoting immune response and shows antioxidant activity (48, 49). Further, L-arginine could boost liver detoxification and alleviate the hepatotoxicity induced by CCl4 in mice (50). Glycine is one of the three amino acid constituents of glutathione, which is one of the most important antioxidants (51). Busulfan could dissipate a large quantity of glutathione in mitochondria, leading to oxidative stress (52, 53). The significantly increased level of glycine in the PBA.1 and PBA1 groups could be beneficial for the recovery of damage caused by busulfan. We also observed that 14,15-DiHETrE in tetrahydrofurandiol pathway was significantly downregulated in all the RAAE treatment groups. Tetrahydrofurandiols have been found to significantly disturb sexual behavior and fertility in male rats (54, 55). Therefore, the downregulation of 14,15-DiHETrE might indirectly contribute to the recovery of spermatogenesis in the busulfan-treated mice. In addition, we observed that L-carnitine was upregulated in the busulfan plus RAAE groups compared with busulfan alone. It has been reported that L-carnitine could reduce the side effect of busulfan-induced damage on the reproductive system of male rats (37). L-carnitine could also improve sperm motility and sperm chromatin quality (56, 57). Furthermore, we detected the downregulation of anandamide after the busulfan treatment and the upregulation of anandamide in PBA1 and PBA5. Lower amount of anandamide has been reported to be associated with reduced sperm mobility and sperm count (58). Taken together, the changes in metabolites, such as LysoPC, L-arginine, glycine, anandamide, and L-carnitine, could all contribute to the positive effect of RAAE on reducing the damage caused by busulfan to the male reproductive system.

## Conclusion

In summary, in this study, we displayed the most abundant content (cyanidin 3-O-galactoside) of RAAE and comprehensively investigated the effects of the RAAE treatment on male reproductive system dysfunction caused by busulfan in mice. When taken together all the analysis of seminiferous tubules, sperm concentration, sperm motility, and plasma metabolism in control and treatment groups, BA1 (busulfan + 1 mg/kg BW) was found to be the best treatment concentration for rescuing spermatogenesis in mice. The study suggests that RAAE could be a potential natural product for the recovery of spermatogenesis after busulfan treatment. However, further study is necessary to analyze the specific anthocyanin component, cyanidin 3-O-galactoside, in improving busulfan-induced damage in mice.

## Data Availability Statement

The data presented in the study are deposited in the EMBL-EBI MetaboLights database with the ID MTBLS2863 and MTBLS2782, and can be accessed through www.ebi.ac.uk/metabolights/MTBLS2782 and https://www.ebi.ac.uk/metabolights/MTBLS2863.

## Ethics Statement

The animal study was reviewed and approved by Animal Care and Use Committee of Qingdao Agricultural University.

## Author Contributions

JX wrote the manuscript. QL revised the manuscript. YZ designed all the experiments. XZ, JX, and XS performed all the experiments and analyzed the data. All the authors approved the final manuscript.

## Conflict of Interest

The authors declare that the research was conducted in the absence of any commercial or financial relationships that could be construed as a potential conflict of interest.

## Supplementary Material

Supplementary Table 1 Number of significantly different metabolites detected in PBA0 vs. control, PBA.1 vs. control, PBA1 vs. control, and PBA5 vs. control, respectively.
